# Large-Area Compatible Laser Sintering Schemes with a Spatially Extended Focused Beam

**DOI:** 10.3390/mi8050153

**Published:** 2017-05-11

**Authors:** Habeom Lee, Jinhyeong Kwon, Woo Seop Shin, Hyeon Rack Kim, Jaeho Shin, Hyunmin Cho, Seungyong Han, Junyeob Yeo, Sukjoon Hong

**Affiliations:** 1 Applied Nano and Thermal Science Lab, Department of Mechanical Engineering, Seoul National University, 1 Gwanak-ro, Gwanak-gu, Seoul 151-742, Korea; habeom.lee@snu.ac.kr (H.L.); jhs0909k@snu.ac.kr (J.K.); jayz.shin84@gmail.com (J.S.); augustinus310@snu.ac.kr (H.C.); 2 Department of Mechanical Engineering, Hanyang University, 55 Hanyangdaehak-ro, Sangnok-gu, Ansan Gyeonggi-do 15588, Korea; caribou11@hanyang.ac.kr (W.S.S.); kihll1004@hanyang.ac.kr (H.R.K.); 3 Department of Mechanical Engineering, Ajou University, San 5, Woncheon-Dong, Yeongtong-Gu, Suwon 16499, Korea; sy84han@ajou.ac.kr; 4 Novel Applied Nano Optics (NANO) Lab, Department of Physics, Kyungpook National University, 80 Daehak-ro, Bukgu, Daegu 41566, Korea

**Keywords:** laser sintering, metal nanoparticle, micro lens array, cylindrical lens, flexible electrode

## Abstract

Selective laser sintering enables the facile production of metal nanoparticle-based conductive layers on flexible substrates, but its application towards large-area electronics has remained questionable due to the limited throughput of the laser process that originates from the direct writing nature. In this study, modified optical schemes are introduced for the fabrication of (1) a densely patterned conductive layer and (2) a thin-film conductive layer without any patterns. In detail, a focusing lens is substituted by a micro lens array or a cylindrical lens to generate multiple beamlets or an extended focal line. The modified optical settings are found to be advantageous for the creation of repetitive conducting patterns or areal sintering of the silver nanoparticle ink layer. It is further confirmed that these optical schemes are equally compatible with plastic substrates for its application towards large-area flexible electronics.

## 1. Introduction

A metal electrode layer, either patterned or in a thin-film form, is an inevitable component of an electronic device. Fabrication of a metal electrode layer based on conventional photolithographic means with vacuum evaporation techniques has achieved tremendous success with silicon-based electronics thus far, however, it has often been found to be improper for flexible and stretchable electronics. New types of conductive layers based on metal nanoparticle (NP) ink have provided a possible solution to this problem. As the melting temperature of metal NPs decreases according to its diameter due to the size effect, [1,2] a metal electrode layer is easily created on an arbitrary substrate at a mild temperature in ambient conditions through the direct coating and sintering of metal NP ink. However, despite its advantages, the minimum feature size of the resultant electrode prepared by metal NPs was relatively coarse due to the limited resolution of the conventional printing techniques which were employed for the selective deposition of metal NP ink [3]. At the same time, the sintering scheme remained questionable for a number of heat-sensitive substrates, as it was experimentally verified that the conventional sintering step with substantial sintering time can damage the underlying substrate [4].

As an alternative to the conventional sintering scheme, selective laser sintering of metal NPs was introduced to conduct patterning and sintering of metal NP ink simultaneously using a focused laser beam [5,6]. The focused laser beam is employed as a localized heat source based on the photothermal reaction, [7] and the area subject to heating is mainly determined by the spatial intensity distribution of the focused laser beam. Yeo et al. [8] demonstrated that the feature size of the resultant electrode can be easily reduced down to several micrometers using a laser beam focused by a telecentric lens module, which is difficult to achieve with other printing techniques. In addition, it was confirmed that the thermal damage on the underlying substrate can be minimized owing to the reduced heat-affected zone generated by a focused laser. These results suggest that laser sintering can be a convenient technique for the creation of metal patterns on flexible substrates. At the early stage, noble metal NPs such as gold (Au) [5,6,9,10,11,12,13,14,15] and silver (Ag) NP ink [1,2,4,8,16,17,18,19,20,21,22,23] were employed as the target materials for laser sintering, but recent studies demonstrate that the application of laser sintering can be extended to other oxidation-sensitive metal NPs such as copper (Cu) [24,25,26], even in ambient conditions, by reducing the local heating time through rapid scanning of the focused laser beam. 

Laser sintering, however, is often considered to be inappropriate for mass production since it is a direct writing method in principle. As the electrode pattern becomes denser, the time required for the scanning increases linearly. At the same time, a thin-film electrode is another form of electrode that is difficult to be manufactured by the laser sintering method. Laser sintered thin-film electrodes not only require raster scanning of the entire area, but also often show imperfect electrical conductivity due to the discontinuities in electrical path originated from separate scanning steps. In this study, we introduce extended laser sintering schemes with a spatially modified focused beam to assist in the facile production of the electrodes with denser patterns or thin-film metal layers. In detail, a focusing lens is substituted by a micro lens array (MLA) and a cylindrical lens, designated for the fabrication of densely patterned electrodes and thin-film metal layers, respectively. It is also confirmed that the proposed optical schemes are still compatible with plastic substrates for their application in large-area flexible electronics.

## 2. Materials and Methods

Ag NP ink is firstly synthesized with the two-phase method, as reported in previous studies [8,17]. The resultant Ag NP is ~5 nm in diameter and encapsulated with self-assembled monolayer (SAM) to prevent agglomeration between NPs. It is confirmed that the Ag NP ink experiences melting when the temperature reaches ~150 °C, which is significantly lower than its bulk counterpart, due to the melting point depression from the size effect [2]. The synthesized Ag NP ink is coated on arbitrary substrates by spin-coating at 1000 rpm, and dried in a convection oven at 70 °C for 3 min to evaporate the excessive solvent. The target substrate can be either a rigid substrate such as a silicon wafer or a slide glass, or a flexible substrate. Polyimide (PI) thin film with a thickness of 150 μm is selected as the flexible substrate throughout the study.

**Figure 1:**
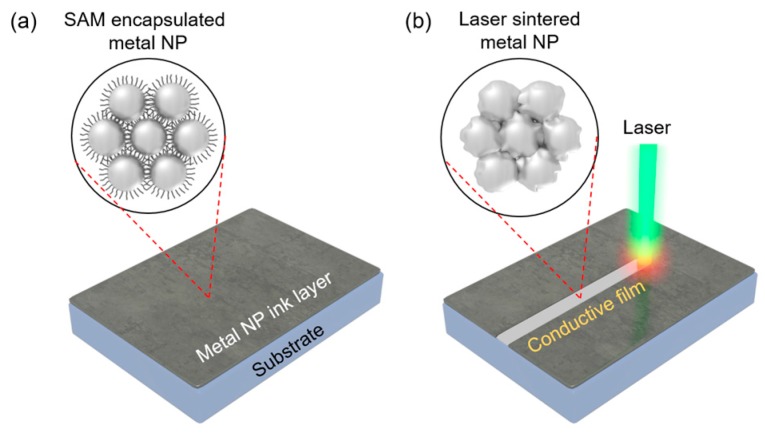
Schematic illustration of metal nanoparticle (NP) ink layer on a substrate (**a**) before and (**b**) after laser sintering.

A thin-film composed of Ag NPs is prepared on the substrate after the coating and drying steps, as shown in Figure 1a. Although Ag NPs are closely packed together, the as-prepared NP ink layer does not exhibit good electrical conductivity since the Ag NPs exist as separate entities. These Ag NPs can be transformed into a continuous conductive layer once a focused laser beam is scanned along the designated path, as shown in Figure 1b. The local temperature of the area subject to the laser irradiation increases rapidly and the Ag NPs experience melting and solidification steps as a consequence. The Ag NPs after the laser scanning subsequently show different physical properties from the as-deposited area, such as reflective color and high electrical conductivity.

**Figure 2:**
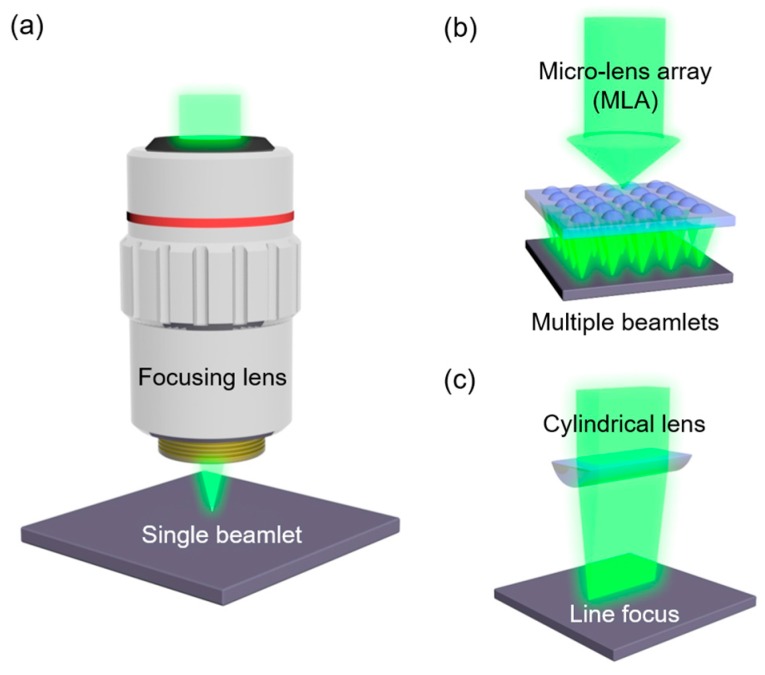
Optical schemes for selective laser sintering with (**a**) a single beamlet, (**b**) multiple beamlets, and (**c**) a focal line.

An optical system is required to focus the laser beam on a designated spot, and an objective lens is frequently employed for the focusing, as shown in Figure 2a. The scanning path is controlled by a programmable motorized stage. Instead of moving the stage, a galvano-mirror together with a telecentric lens can be employed to achieve fast scanning of the laser beam over a large area [8,17]. Together with single focusing scheme, an MLA and cylindrical lens have been exploited in this study as new optical schemes for large-area compatible laser sintering, as depicted in Figure 2b,c. For single focusing, a green wavelength continuous wave laser (Millenia V, Spectra-Physics, Santa Clara, CA, USA) is scanned by a 2D galvano-mirror scanning system (hurrySCAN II, Scanlab GmbH, Puchheim, Germany) while the laser is focused by an f-theta telecentric lens with f = 100 mm. The laser scanner system is controlled by a computer with CAD software (SAMLight, SCAPS GmbH, Oberhaching, Germany) to draw arbitrary patterns. For the generation of multiple beamlets, an MLA with 400–900 nm anti-reflective coating is employed together with a 5× objective lens. More detailed information on the optical system is included in the Results and Discussions section. The MLA has a pitch of 300 µm and a focal length of 18.6 mm. For a focal line, the MLA is replaced by a plano-convex cylindrical lens with f = 50 mm. The laser scanning speed is fixed at 5 mm/s in every case.

## 3. Results and Discussion

**Figure 3:**
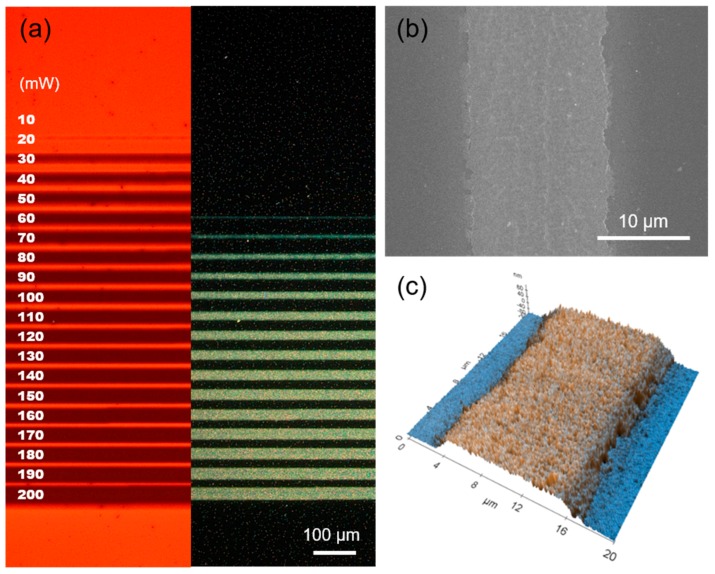
Laser sintering results by a single beamlet. (**a**) Optical images of Ag NP ink sintered at different laser power. (Left) Bright field; (Right) Dark field images. (**b**) Scanning electron microscope (SEM) image of the laser-sintered Ag NP at 100 mW laser power, and (**c**) its atomic force microscopy (AFM) image.

Figure 3 shows the sintering results created by the 2D galvano-mirror scanning system with the single focusing beamlet on a glass substrate in terms of their optical, scanning electron microscope (SEM) and atomic force microscopy (AFM) images. More information on the optical setting can be found in the previous studies [8,17]. Laser sintering is firstly conducted on an Ag NP ink layer according to the irradiated laser power. Figure 3a shows the optical microscope image of the Ag NP ink layer after the scanning, whereas the left and the right columns are the bright and dark field images of the same region, respectively. From the bright field image, it is noticeable that the optical transmission starts to change at the laser power of ~20 mW, while the dark field image shows no difference until the power reaches ~60 mW. Electrical measurement reveals that the resultant conductor line does not exhibit substantial electrical conductivity until it becomes reflective in the dark field image. We therefore anticipate that the change in optical transmission in the bright field image is due to the evaporation of the trapped solvent, or from the incomplete sintering between a small fraction of Ag NPs. The width of the area affected by the laser sintering is slightly bigger in the bright field image, and it is highly dependent to the drying condition.

Figure 3b is the SEM image of the laser-sintered Ag NP at 100 mW laser power after the removal of remaining Ag NP ink from the substrate. The area irradiated by the laser remains on the substrate as a thin electrode while the other Ag NPs are washed away. More detailed information about its 3D morphology can be confirmed from its AFM image in Figure 3c. The height of the resultant conductor is ~120 nm, and this value can be further controlled by changing the wet processing conditions including spin-coating speed and drying conditions [17]. 

**Figure 4:**
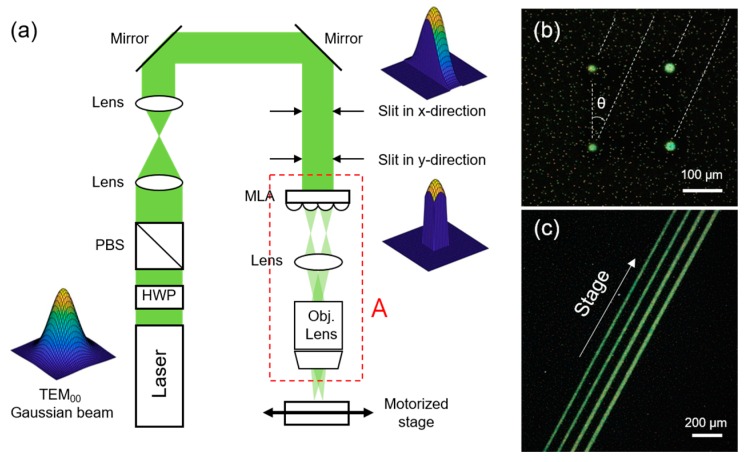
Laser sintering results by multiple beamlets. (**a**) Optical setting for multiple beamlets and a focal line (HWP: Half-wave plate, PBS: polarized beam splitter, MLA: Micro lens array). (**b**) Optical image of Ag NP ink by sintering with the multiple beamlets at a single exposure and (**c**) after moving the stage at a slanted angle.

In order to generate a spatially extended focused beam, we first modified the optical setting, as shown in Figure 4a. Since our objective is to split the incident beam into multiple beamlets or to extend the focus into a focal line, incident laser intensity becomes an important issue. Two achromatic lenses with different focal lengths have been added in a Keplerian telescope configuration in order to reduce the beam size for sufficient laser intensity. At the same time, two adjustable slits are installed in the *x*- and *y*- directions to cut the incident laser beam, since a flat-top intensity profile is demanded in large-area applications instead of a TEM_00_ (Fundamental Transverse Mode) Gaussian beam to ensure spatial uniformity in processing. The truncated laser beam shows a relatively flat-top intensity profile, but it is not quite perfect and other optical components such as a homogenizer will be required for further advancement of the proposed technique [18].

Figure 4b is the optical image of the Ag NP ink layer after a single exposure to the multiple beamlets. The multiple beamlets are generated by opening each slit at a width of 600 μm, so that the incident beam covers 2 × 2 = 4 cells in the 300-μm pitch MLA. Different from the single beamlet case, four distinct spots are transformed into the conductive electrode with a single exposure as confirmed from the reflective color on the dark field optical image. Their relative positions can be further controlled by changing the parameters in the optical setting. Using the multiple beamlets, repetitive patterns can be easily created. As a representative example, parallel conductive lines, which are basic components for various applications including wire-grid polarizer and grid-type transparent conductors, [17] are created by moving the stage at a slanted angle. Figure 4c shows the resultant parallel lines produced by a single translational movement of the motorized stage.

**Figure 5:**
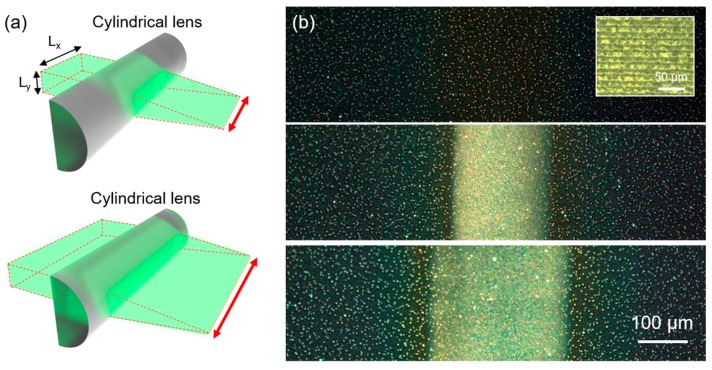
(**a**) Schematic illustration of the laser sintering by a focal line. (**b**) The optical image of the resultant Ag NP ink after scanning at (Top) *L_x_* = 50 μm, (Middle) *L_x_* = 150 μm and (Bottom) *L_x_* = 250 μm (Inset: Ag NP thin-film conductor created by raster scanning of a single beamlet).

For the sintering of metal NP ink over the entire area as a thin-film conductor without any patterns, a focal line is more suitable compared to the multiple beamlets. The optical components in Figure 4a (denoted as “A”) are replaced by a cylindrical lens to create a focal line. In the current configuration, slit width in the *y*- direction (*L_y_*) determines the laser intensity at the focus, while the slit width in the *x*- direction (*L_x_*) controls the length of the focal line, as shown in Figure 5a. In this experiment, *L_y_* has been fixed at ~300 μm throughout the study, while *L_x_* is altered from 50 μm to 250 μm. Although the intensity of the incoming laser remains the same, it is found that the sintering characteristics of the resultant Ag NP ink are different in each case, as shown in Figure 5b. We estimate that the inconsistency comes from the different heat transfer conditions. For an extended focal line, an equal amount of heat is generated along the line and hence the major heat dissipation should occur towards a perpendicular direction to the focal line. However, as the length of the focal line becomes smaller, heat starts to dissipate in every lateral direction so that the resultant temperature reached by a shorter focal line should be lower at *L_x_* = 50 μm. Figure 5b shows that the Ag NP ink at a width of >150 μm is successfully sintered by a single scanning with the cylindrical lens. It is worth mentioning that the resultant conductive layer does not have apparent boundaries which can be found in the thin-film conductors produced by raster scanning of a single beamlet, as shown in the inset.

**Figure 6:**
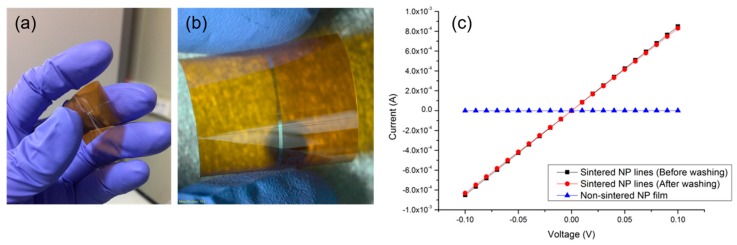
Application of the proposed optical schemes for laser sintering of Ag NP ink on a flexible substrate. Optical image at (**a**) low magnification and (**b**) high magnification; (**c**) current-voltage (IV) curve of the resultant Ag electrode on the flexible substrate.

In order to verify that the proposed optical schemes are equally compatible with flexible substrates, parallel Ag NP lines are created on a flexible PI substrate with the modified optical setting. It is apparent from Figure 6a,b that consistent lines are created on the PI substrate without any apparent thermal damage. The current-voltage (IV) curve of the resultant conductor is measured to ensure that their electrical characteristics are suitable for the application of flexible electronics. The IV curve in Figure 6c shows that the sintered lines exhibit ohmic behavior and the calculated resistivity is estimated to be 7~8 times higher than bulk Ag, while the as-prepared NP film displays insignificant electrical conductivity. 

In summary, we propose two forms of a spatially extended focused beam intended for the fabrication of Ag NP conducting layers with dense patterns or in thin-film configuration. It is demonstrated that the modified optical setting can produce multiple beamlets for repetitive patterns or focal lines for thin-film conductors. Since these optical schemes are equally compatible with flexible substrates, it is expected that the proposed techniques will supplement fabrication of large-area flexible electronics.
